# Successful Treatment of Cardiac Tamponade due to Rupture of the Heart Performing an Open-chest Pericardiotomy

**DOI:** 10.7759/cureus.7101

**Published:** 2020-02-25

**Authors:** Shoichiro Yatsu, Youichi Yanagawa, Hiroki Nagasawa, Keiichi Tambara, Satoru Suwa

**Affiliations:** 1 Cardiology, Juntendo University Shizuoka Hospital, Izunokuni, JPN; 2 Acute Critical Care Medicine, Juntendo University Shizuoka Hospital, Izunokuni, JPN; 3 Cardiovascular Surgery, Juntendo University Shizuoka Hospital, Izunokuni, JPN

**Keywords:** tamponade, cardiac arrest, pericardiotomy

## Abstract

A 78-year-old woman with mild dementia was found unconscious by her family. She was transported by an ambulance to our emergency room (ER). Initially, she was comatose and in a state of shock. The echocardiographic findings suggested cardiac tamponade by hematoma. Computed tomography also showed tamponade without aortic dissection. After imaging, she went into cardiac arrest, was returned to the ER, and tracheal intubation and left thoracotomy for pericardiotomy were performed. A return of spontaneous circulation was obtained by following this procedure. Bleeding from a rupture of the left cardiac free wall was confirmed, and the rupture was closed with TachoSil®. After closing the thoracotomy, electrocardiography revealed ST elevation in the precordial leads. Subsequently, placement of an indwelling intra-aortic balloon pump and coronary angiography (CAG) were performed. CAG showed an occlusion of the anterior interventricular branch and circumflex branch of the left coronary artery. She underwent conservative therapy in a coronary care unit. Finally, after obtaining hemodynamic stability and baseline mental status, she was transferred to another medical facility.

We herein report a rare case involving the successful treatment of cardiac tamponade due to rupture of the heart performing an open-chest pericardiotomy and additionally discuss the key points for obtaining a favorable outcome.

## Introduction

Rupture of the left ventricle free wall is a catastrophic complication in patients suffering from acute myocardial infarction (AMI). In the reperfusion era, its incidence is approximately 3% and accounts for 12% mortality and up to 60% of in-hospital deaths in patients presenting in a state of shock [1]. Most patients succumb to rapid deterioration and instantaneous death; few have a less acute course that leads to a series of diagnostic procedures with subsequent intervention. Survivors of this catastrophic event have not been frequently reported [2-4]. We herein report a case of cardiac tamponade due to rupture of the left ventricle free wall in which a favorable outcome was obtained by open-chest pericardiotomy.

## Case presentation

**Figure 1:**
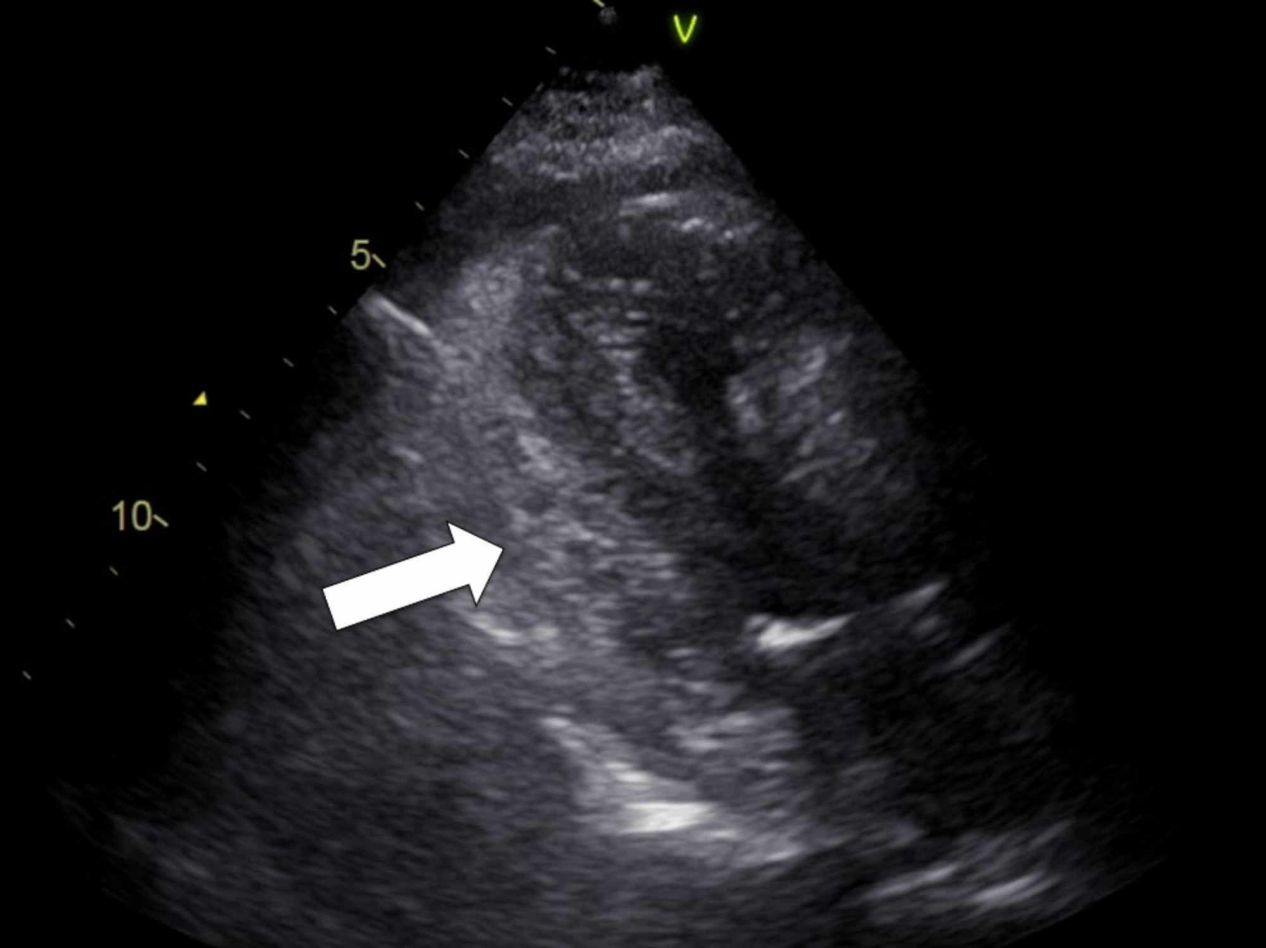
Ultrasound on arrival The echocardiographic findings suggest cardiac tamponade by hematoma (arrow: hyperechoic area). The heart was compressed by hematoma.

A 78-year-old woman with mild dementia was found unconscious by her family. She was transported by an ambulance to our emergency room (ER). She was independent in her activities of daily living. She was prescribed donepezil, risperidone and Yokukansan. On arrival, she presented with a Glasgow Coma Scale of 6 (E1V1M4). Initially, her blood pressure was 56/44 mmHg and her heart rate was 76 beats per minute. The echocardiographic findings suggested cardiac tamponade due to an intrapericardial hematoma (Figure 1).

**Figure 2:**
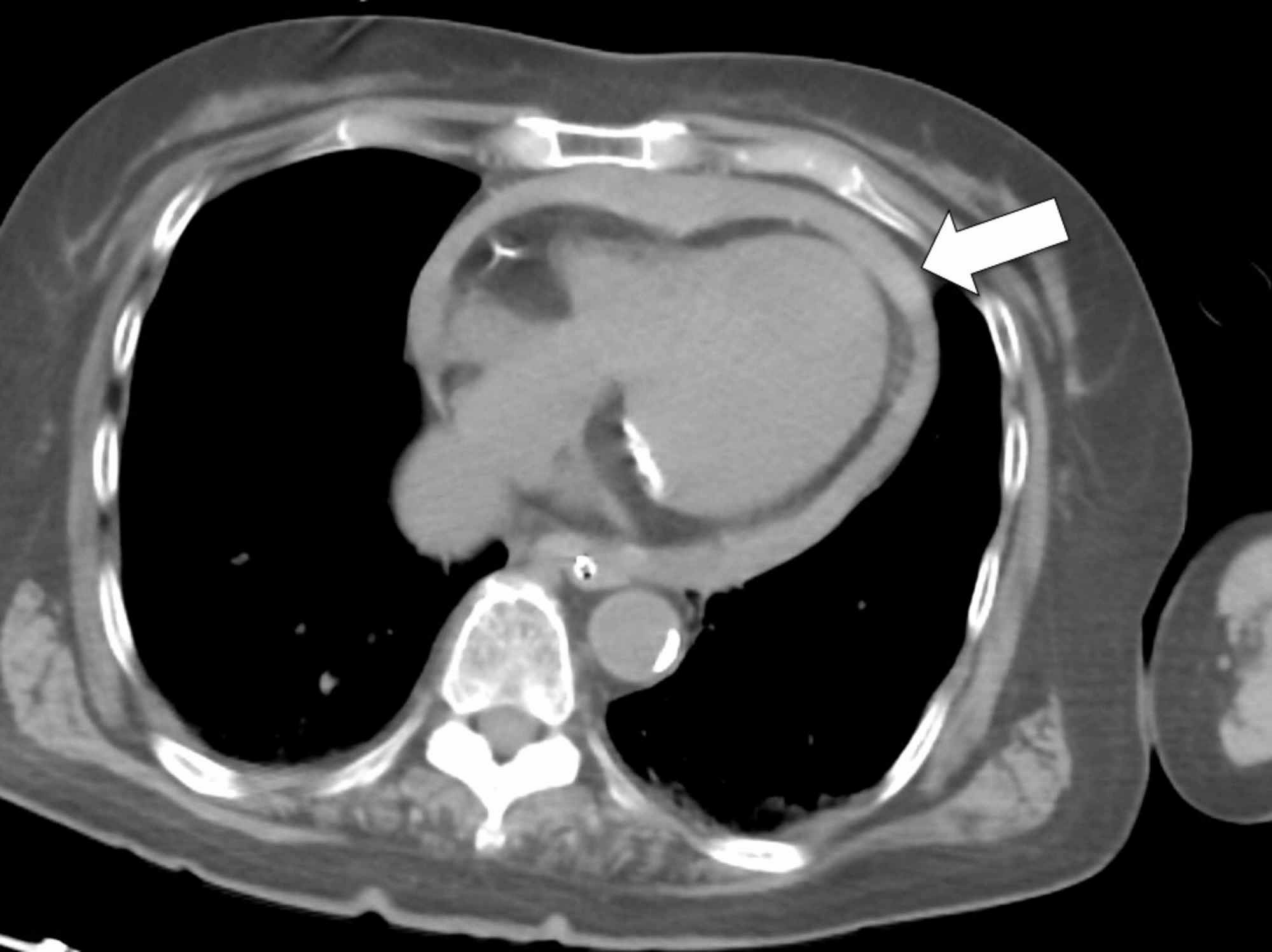
Computed tomography (CT) on arrival CT demonstrated cardiac tamponade (arrow).

To exclude aortic dissection, she underwent computed tomography (CT). CT also showed cardiac tamponade without aortic dissection (Figure 2).

**Figure 3:**
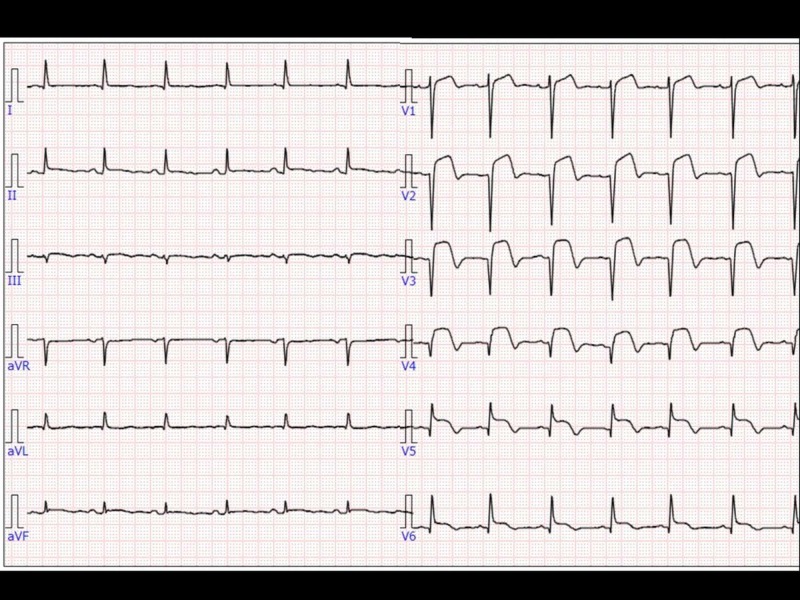
Electrocardiogram (ECG) after closing thoracotomy ECG showed ST elevation in the precordial leads.

Following this examination, she went into cardiac arrest. She was immediately returned to the ER, and underwent tracheal intubation and a left thoracotomy to perform a pericardiotomy. A return of spontaneous circulation was achieved following this procedure. Bleeding from a rupture site in the left cardiac free wall was confirmed, which was closed with TachoSil® (a tissue sealant matrix; CSL Behring, Tokyo, Japan). The rupture site demonstrated an oozing type of bleeding [5]. After closing the thoracotomy with the placement of a chest drainage tube, electrocardiography showed ST elevation in the precordial leads (Figure 3).

**Figure 4:**
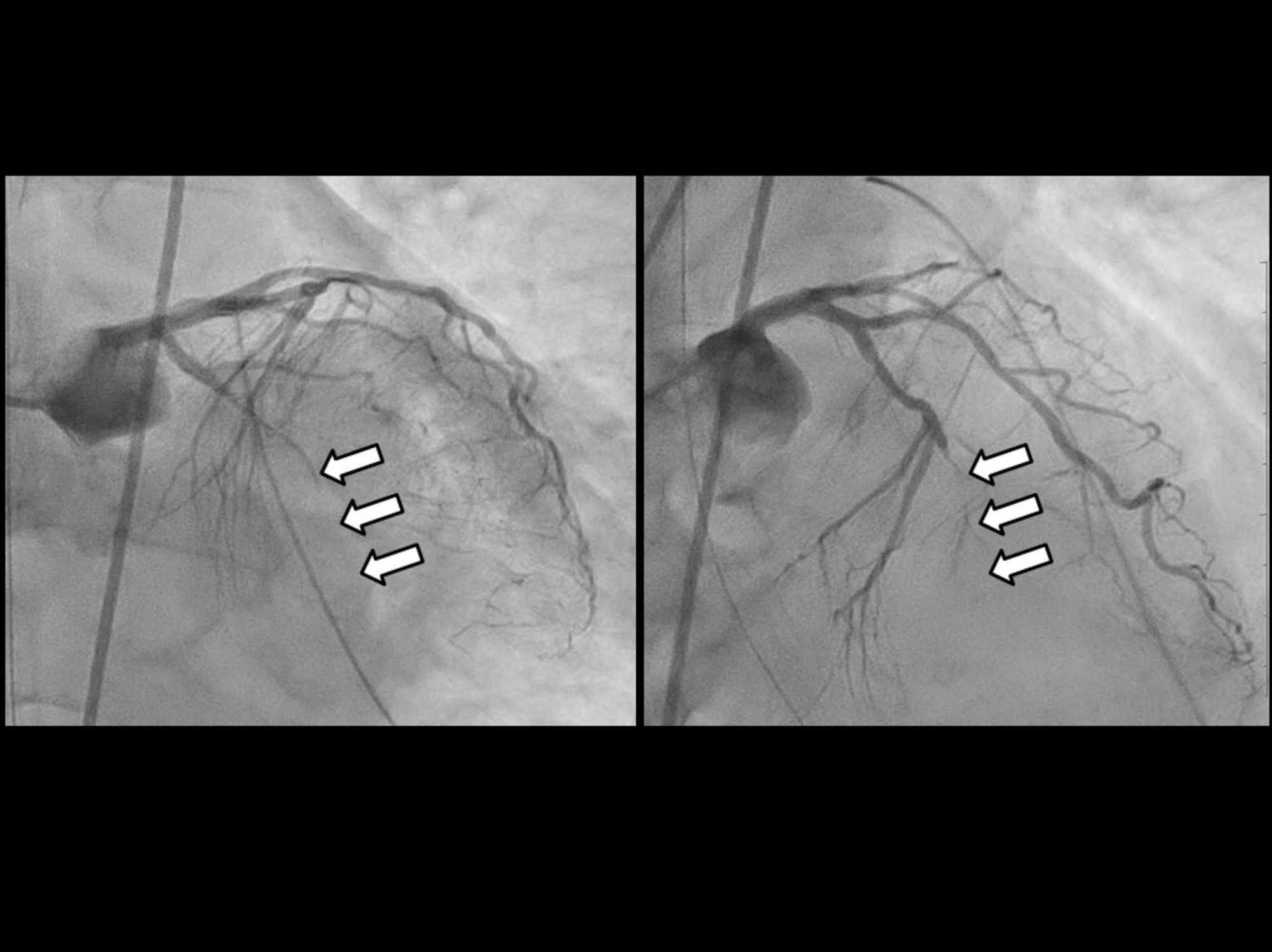
Coronary angiography (CAG) on arrival CAG showed an occlusion of the anterior interventricular branch (segment 7: left) and circumflex branch (segment 13: right) of the left coronary artery.

Subsequently, she underwent placement of an indwelling intra-aortic balloon pump (IABP) and coronary angiography (CAG). CAG showed an occlusion of the anterior interventricular branch (segment 7) and circumflex branch (segment 13) of the left coronary artery with collateral blood supply (Figure 4).

Her family did not consent to a radical heart operation; thus, she received conservative therapy in a coronary care unit. She regained a stable hemodynamic state and baseline mental status, and the IABP was removed on the third hospital day. Tracheal intubation and the chest drainage tube were removed on the seventh hospital day. Thereafter, she was transferred to another medical facility for rehabilitation.

## Discussion

We reported an extremely rare case in which cardiac tamponade with cardiac arrest due to the rupture of the heart was successfully treated using open-chest pericardiotomy with patching of the rupture site with TachoSil®.

Left ventricular rupture is one of the most serious mechanical complications of AMI [6,7]. Left ventricular free wall rupture is an infrequent complication (0.2%-7.6%), but it is associated with a high mortality due to pericardial tamponade [6,7]. Patients with left ventricular rupture have been shown to have a significantly lower incidence of previous MI, previous percutaneous coronary intervention, and hypertension [6]. Furthermore, older age and female gender are significantly associated with left ventricular rupture, similar to the findings in our case report [6]. The following characteristics are associated with in-hospital mortality in a univariable analysis according to Formica et al.: pre-existing hypertension, need for inotropes and cardiac arrest at presentation, cardiopulmonary resuscitation, preoperative extracorporeal membrane oxygenation, repair of a left ventricular wall rupture site, operation on extracorporeal membrane oxygenation, and postoperative extracorporeal membrane oxygenation [7]. In a multivariable analysis, cardiac arrest at presentation was an independent predictor of in-hospital mortality [7]. Accordingly, due to the severe outcome of left ventricular free wall rupture after AMI with an accompanying cardiac arrest, only two cases in the English literature and one in the Japanese literature have reportedly obtained a survival outcome [8-10]. The two previous reports, which documented the occurrence and treatment of a left ventricular free wall rupture with cardiac arrest, contained no detailed individual information [6,7]. We summarized these three case reports in Table 1.

**Table 1:** List of survival outcome of left ventricular free wall rupture after acute myocardial infraction with cardiac arrest

No.	Reporter [reference number]	Age (years)	Sex	Treatment
1	Okabe [8]	59	F	Pericardiocentesis, bovine pericardial patch with suturing, bypass grafting by open-chest surgery
3	Walts [9]	71	M	Cardiopulmonary bypass, bovine pericardial patch with suturing under open-chest surgery
3	Kagaya [10]	70	F	Percutaneous cardiopulmonary support, hydrofit, bioglue, and TachoSil® under open-chest surgery

Three key points must be considered when treating cardiac tamponade due to rupture of the heart. First, pericardiocentesis may not be effective for the resolution of hemoperricardium due to the presence of a thrombus, in which pericardiocentesis would not be effective [1,4,11,12]. Accordingly, urgent pericardiotomy is necessary if pericardiocentesis cannot retrieve blood within the pericardial sac causing hemotamponade. Second, a surgical procedure is necessary to repair the rupture site. Accordingly, an open-chest pericardiotomy is useful not only for the release of hemotamponade but also for the detection of the rupture site. Third, applying TachoSil® alone may be effective for repairing a rupture of the heart. Okamura et al. reported consecutive patients who underwent sutureless repair using TachoSil® without cardiopulmonary bypass, with an overall survival at 10 years of 63% [5]. Applying TachoSil® alone to the rupture site is a simple method of treatment. However, it should be noted that two patients with significant left ventricular wall ruptures experienced re-rupture. Accordingly, TachoSil® may not be suitable for the treatment of blow-out type rupture.

## Conclusions

We reported an extremely rare case in which cardiac tamponade with cardiac arrest due to rupture of the heart was successfully treated by open-chest pericardiotomy and discussed the key points in obtaining a favorable outcome.
